# Co-synergism of endophyte *Penicillium resedanum* LK6 with salicylic acid helped *Capsicum annuum* in biomass recovery and osmotic stress mitigation

**DOI:** 10.1186/1471-2180-13-51

**Published:** 2013-03-01

**Authors:** Abdul Latif Khan, Muhammad Waqas, Muhammad Hamayun, Ahmed Al-Harrasi, Ahmed Al-Rawahi, In-Jung Lee

**Affiliations:** 1 Department of Biological Sciences and Chemistry, University of Nizwa, Nizwa, Oman; 2 School of Applied Biosciences, Kyungpook National University, Daegu, Republic of Korea; 3 Kohat University of Science & Technology, Kohat, Pakistan; 4 Department of Botany, Abdul Wali Khan University, Mardan, Pakistan

**Keywords:** *Penicillium resedanum LK6*, Osmotic stress, Salicylic acid, Antioxidants, Biomass recovery, *Capsicum annuum* L

## Abstract

**Background:**

Water-deficiency adversely affects crop growth by generating reactive oxygen species (ROS) at cellular level. To mitigate such stressful events, it was aimed to investigate the co-synergism of exogenous salicylic acid (SA) and symbiosis of endophytic fungus with *Capsicum annuum* L. (pepper).

**Results:**

The findings of the study showed that exogenous SA (10^-6^ M) application to endophyte (*Penicillium resedanum LK6*) infected plants not only increased the shoot length and chlorophyll content but also improved the biomass recovery of pepper plants under polyethylene glycol (15%) induced osmotic stress (2, 4 and 8 days). Endophyte-infected plants had low cellular injury and high photosynthesis rate. SA also enhanced the colonization rate of endophyte in the host-plant roots. Endophyte and SA, in combination, reduced the production of ROS by increasing the total polyphenol, reduce glutathione, catalase, peroxidase and polyphenol oxidase as compared to control plants. Osmotic stress pronounced the lipid peroxidation and superoxide anions formation in control plants as compared to endophyte and SA-treated plants. The endogenous SA contents were significantly higher in pepper plants treated with endophyte and SA under osmotic stress as compared to control.

**Conclusion:**

Endophytic fungal symbiosis and exogenous SA application can help the plants to relieve the adverse effects of osmotic stress by decreasing losses in biomass as compared to non-inoculated plants. These findings suggest that SA application positively impact microbial colonization while in combination, it reprograms the plant growth under various intervals of drought stress. Such symbiotic strategy can be useful for expanding agriculture production in drought prone lands.

## Background

Water-deficient or drought stress conditions can drastically hinder the crop growth and yield. Exposure to extreme conditions brings changes inside plant tissues at ionic/osmotic, phytohormonal and secondary metabolites levels [1]. Continuous waves of drought cause an imbalance in the osmotic potential of the plant tissues, thus, inducing the synthesis of reactive oxygen species (ROS) [2]. To maintain the cellular redox potential and buffer the negative effects of ROS, plant produce antioxidants like reduced glutathione (GSH), total polyphenols, catalase (CAT), peroxidase (POD) and polyphenol oxidase (PPO) etc [3]. Plants tend to accumulate higher antioxidants to avoid cellular damage. Additionally, the plant hormonal apparatus is activated to transduce stress signals during altered osmotic potential. Endogenous salicylic acid (SA) is known to develop defence-related responses during biotic stress [4,5] while exogenous application of SA has mostly showed abiotic stress tolerance for example, heat stress in mustard [6], chilling in maize [7], salinity in wheat [8] and drought in wheat and sunflower [9,10]. Exogenous SA increase shoots length, flowering and yield in various crop plants [4-10]. During pathogenic attack, the endogenous SA in plants is often accumulated whilst the systemic acquired resistance (SAR) is initiated which involve synthesis of *pathogenesis-related* (*PR*) proteins [3]. Conversely, during interaction with mutualistic plant growth promoting microorganism, it doesn’t involve the synthesis of *PR* protein, thus establishing induced systemic resistance (ISR) [11,12]. In spite of the key role of SA in plant’s defence, its function during endophyte-association has received little attention [13].

Endophytic fungi, residing in the root tissues can play pivotal role in host-plant growth by influencing mineral composition, plant hormonal balance, chemical composition of root exudates, soil structure and plant protection against biotic and abiotic stresses [14-16]. Previous studies have shown that endophytic fungal association can significantly increase plant biomass and growth [14-18]. Studies have also elaborated the beneficial effects of endophytic fungi on the growth responses of host-plants under various stress conditions [15-18]. In present study, we isolated an endophytic fungus, *Penicillium resedanum LK6* from the roots of drought-stressed *Capsicum annuum* (pepper) plants. The endophyte was found to produce various biologically active gibberellins detected in the pure culture through chromatographic techniques and advance spectroscopic analysis (unpublished results). Previous studies also show that some strains of *Penicillium* endophytes can produce gibberellins [17]. Redman et al. [16] and Khan et al. [17] have previously shown that phytohormones producing endophytes/fungi can ameliorate the negative impacts of salinity and drought.

Gibberellins producing fungal endophytes have been envisaged to increase host-plant resistance against salinity, drought, and heat stresses [16,17] however, these are least known for their symbiotic impacts on endogenous and exogenous SA during abiotic stress. Previously, Herrera-Medina et al. [11] explained the influence of exogenous SA on root colonization but it was mostly restricted to arbuscular mycorrhizal fungi [12]. A similar study was reported by Li et al [19] in which the effect of exogenous SA on the colonization of arbuscular mycorrhizal fungi *Glomus mosseae* and growth of *Avena nuda* resistance under NO_2_ exposure were assessed. However, the interaction of exogenous SA and endophyte association with *C. annuum* plants during stress is still poorly understood and unexplored. In present study, it was aimed to understand the co-synergism of SA with endophytic fungus (*Penicillium resedanum LK6*) and its effects on plant biomass recovery under polyethylene glycol (PEG) induced osmotic stress (2, 4 and 8 days).

## Methods

### Growth of endophytic fungus - *Penicillium resedanum LK6*

Approximately, 200 root pieces were collected from *C. annuum* plants growing in water deficient conditions (soil water potential 41.23 hPa). The root pieces were surface sterilized with 2.5% sodium hypochlorite (30 min in shaking incubator at 120 rpm) and washed with autoclaved distilled water (DW) to remove the contaminants, rhizobacteria and superficial fungi. The pepper root pieces (about 0.5 cm) were kept in petri-plates containing Hagem medium (0.05% NH_4_Cl, 0.1% FeCl_3_, 0.05% KH_2_PO_4_, 0.5% glucose, 0.05% MgSO_4_.7H_2_O, 1.5% agar and 80 ppm streptomycin; pH 5.6 ± 0.1). The sterilized roots pieces were imprinted to ensure the effectiveness of sterilization process Redman et al. [16]. The emerging fungal spots from the root pieces were isolated and transferred to Potato Dextrose Agar (PDA) medium under aseptic conditions. Among isolated endophytes, a bioactive strain was selected through screening bioassays using dwarf mutant and normal cultivars of *Oryza sativa.* The endophyte was identified by DNA extraction, PCR techniques, sequencing and phylogenetic analysis of Internal Transcribed Spacer [ITS-1 (5^′^-TCC GTA GGT GAA CCT GCG G-3^′^) and ITS-4 (5^′^-TCC TCC GCT TAT TGA TAT GC-3^′^)] with the method previously described by Redman et al. [16] and Khan et al. [17]. The sequence of the endophyte (*P. resedanum*) was submitted to GenBank and was given accession no. JX111908. The endophyte was inoculated in Czapek broth (1% peptone, 1% glucose, 0.001% FeSO_4_.7H_2_O, 0.05% MgSO_4_.7H_2_O, 0.05% KCl; pH 7.3 ± 0.2) and incubated for 10 days at 28°C under shaking (150 rpm) conditions to undertake further experiments [17,18].

### *C. annuum* growth with endophyte

The *C. annuum* seeds were sterilized with 2.5% sodium hypochlorite for 30 min, and rinsed with autoclaved DW. Seeds were incubated in darkness for 24 h to obtain equally germination. The pre-germinated seeds were cultivated in autoclaved pots (121°C for 15 min; two times; 10 × 5 cm) with substrate (peat: perlite: vermiculite – 1:1:1 by volume). The endophyte was cultured in Czapek broth containing conidia (20 ml with 25 propagules/pot) and added to substrate as described previsouly [16-18]. The control plants only received 20 ml/pot of endophyte-free Czapek broth. Thus, pre-germinated pepper seeds and endophyte were grown together for three weeks in the growth chamber (day/night cycle: 14 h; 28°C/10 h; 25°C; relative humidity 60–70%; light intensity 1000 μEm^-2-^s Natrium lamps) irrigated with distilled water.

### Drought stress, endophyte association and SA treatments

The experiment was conducted with a completely randomized block design. Salicylic acid (SA-10^-6^ M) was exogenously applied to pepper plants. The treatments included (i) control, (ii) control plants under drought stress, (iii) plants with endophyte (EA), (iv) EA plants under stress, (v) SA-treated plants, (vi) SA-treated plants under stress, (vii) SA and endophyte-infected plants and (viii) SA and endophyte-associated plants under stress (SA+EA). Each treatment contained 18 plants and the experiment was repeated three times. Drought stress was initiated by exposing plants to 15% polyethylene glycol (PEG 10,000 MW; -3.02 MPa osmotic potential) for 2, 4 and 8 days. The growth parameters i.e. shoot length and fresh weights were measured at harvest while chlorophyll content of leaves was measured by chlorophyll meter (SPAD-502 Minolta, Japan). All readings were taken in triplicate.

The effect on the plant biomass was measured after endophyte and SA treatments under different stress regimes [18]. The biomass gained/lost in endophyte-inoculated and non-inoculated plants were compared by using this formula:

$$\mathrm{Biomass}\;\mathrm{increment}{:}^{\mathrm{DW}\left(E+\right)–\mathrm{DW}\left(E-\right)}{/}^{\mathrm{DW}\left(E-\right)}\times 100.$$

DW is the dry weight while E+ and E- are plants with or without endophyte infestation respectively.

### Determination of electrolytic leakage

Electrolytic leakage was determined according to the method of Liu et al. [20]. Briefly, fresh leaf samples (200 mg) were cut into 5 mm small pieces length and placed in test tubes containing 10 ml DW. The preliminary electrical conductivity (EC_1_) was measured after the tubes were kept in water bath at 25°C for one hour. The samples were autoclaved at 121°C for 20 min to completely kill the tissues and release all electrolytes from leaf tissues. When the samples were cooled down to 25°C, final electrical conductivity (EC_2_) was measured. The electrolyte leakage (EL) was estimated using formula: EL = EC_1_/EC_2_.

### Microscopic analysis and colonization

Plant roots infected with fungal endophyte were sectioned and treated with sodium hypochlorite (2.5%) for 10 min for clarification. Latter, it was treated with KOH (20%) for 24 h which was extensively rinsed with autoclaved DW. The root pieces were acidified with HCl (10%); stained for 24 h using tryptophan blue (0.8%) and lactic acid (95%). At the end, the root pieces were distained in lactic acid for 24 h. The endophytic colonization in roots pieces was assessed through light microscope (Stemi SV 11 Apo, Carl Zeiss). The rate of colonization was determined according to the method of Kumar and Hyde [21].

### Determination of antioxidants

To determine reduced glutathione (GSH), leaves tissues (100 mg) of all the treated pepper plant samples were ground in 3 ml 5% (v/v) trichloroacetic acid using chilled mortar and pestle. The homogenate was obtained through centrifugation (at 15000 rpm for 15 min at 4°C). The homogenate obtained was analysed for reduced glutathione (GSH) activity as described by Ellman [22]. The reaction mixture comprised of sample supernatant (0.1 ml), monosodium phosphate (3.0 ml; 150 mM NaH_2_PO_4_; pH 7.4) and Ellman’s reagent (0.5 ml). The mixture was incubated at 30°C for 5 min. Absorbance was determined at 412 nm and the GSH activity was calculated by a standard curve.

Total polyphenol content was determined by the Folin-Ciocalteau method as mentioned by Kumazawa et al. [23]. Plant tissues (100 mg) were ground with 80% ethanol and the resultant extracts (0.5 ml) were mixed with Folin-Ciocalteau reagent (0.5 ml) and 10% Na_2_CO_3_ (0.5 ml). The absorbance of the reaction mixture was measured at 760 nm after 1 h incubation at room temperature. Total polyphenol content was expressed as micro g/mg (gallic acid equivalents).

The detection of superoxide anion (O_2_^-^) was based on its ability to reduce nitro blue tetrazolium (NBT) as performed by Doke [24]. Treated plant tissues (100 mg) were cut into 1 mm^2^ pieces and immediately immersed in 10 mM phosphate buffer (pH 7.8), containing NBT (0.05% (w/v)) and 10 mM NaN_3_. The reaction mixture was left for incubation till one hour at room temperature. The reaction mixture was heated at 85 ± 2°C for 15 min and cooled quickly to 0°C. The absorbance was measured at 580 nm. The O_2_^-^ content was expressed as an increase of absorbance / 0.1 g dry weight.

The extent of lipid peroxidation was determined by the method of Ohkawa et al. [25]. The optical density of the resulting light pink colour was recorded at 532 nm. Tetramethoxypropane was used as an external standard. The level of lipid peroxides was expressed as micro moles of malondialdehyde (MDA) formed/g tissue weight.

### Enzymatic analysis

All treated plant’s leaves (200 mg) were homogenized in 50 mM Tris–HCl buffer (pH 7.0) composed of 3 mM MgCl_2_, 1 mM EDTA and 1.0% PVP and then centrifuged (15,000 rpm for 15 min at 2°C). The supernatant was used for enzymatic analysis. All parameters were expressed as activity per mg protein. Total proteins were determined according to Bradford [26] method.

Catalase (EC 1.11.1.6) activity was measured as describe by Aebi [27]. The crude enzyme supernatant was treated with 0.2 M H_2_O_2_ (0.5 ml) in 10 mM phosphate buffer (pH 7.0). The enzymatic activity was determined by the decrease in absorbance of H_2_O_2_ at 240 nm. The one unit of catalase is given as μg of H_2_O_2_ released mg protein min^-1^. Peroxidase (EC 1.11.1.7) and polyphenol oxidase (EC 1.14.18.1) activities were measured as described by Kar and Mishra et al. [28] with a little modification. The pepper leaf samples (200 mg) were homogenized with phosphate buffer pH 6.8 (0.1 M) and centrifuged (2°C for 15 min at 17,000 rpm). The clear supernatant was obtained which was analysed for enzymatic activity. The reaction mixture of peroxidase activity composed of 0.1 M phosphate buffer (pH 6.8), pyrogallol (50 μl), H_2_O_2_ (50 μl) and enzyme extract (0.1 ml). After incubation (5 min at 25°C), the reaction was stopped by adding 5% (v/v) H_2_SO_4_ (0.5 ml). The amount of purpurogallin synthesized during the reaction was measured by the absorbance at 420 nm. The same assay mixture, used for peroxidase (without H_2_O_2_), was measured for the activity of polyphenol oxidase. The absorbance of purpurogallin formed was read at 420 nm. One unit of peroxidase and polyphenol oxidase was defined as an increase of 0.1 units of absorbance.

### Endogenous salicylic acid analysis

SA was extracted and quantified as described previously by Seskar et al. [29]. The freeze-dried leaf tissues (0.4 g) of all treated samples were grinded to powder. The powder was sequentially extracted with 90 and 100% methanol by centrifuging (at 15,000 rpm and 4°C). Both the extraction steps were repeated four times until the sample decoloured. The combined methanol extracts were vacuum-dried. Dry pellets were re-suspended in 5% trichloroacetic acid (2.5 ml) while the supernatant was partitioned with ethyl acetate: cyclopentane: isopropanol (100:99:1, v/v). The organic layer containing free SA was transferred to a 4 ml vial and dried with nitrogen gas. The dry SA was rigorously suspended in 1 ml of 70% methanol. High Performance Liquid Chromatography (HPLC) analysis were carried out on Shimadzu coupled with fluorescence detector (Shimdzu RF-10AXL, excitation and emission 305-365 nm respectively) fitted with C18 reverse-phase HPLC column (HP hypersil ODS, particle size 5 μm, pore size 120Å Waters) (Additional file 1: Table S1). The flow rate was 1.0 ml/min. The experiment was repeated three times.

### Statistical analysis

The eight different treatments comprised of eighteen plants per treatment while the experiments were performed in triplicate. The mean, standard error and the graphical representation was done using Graph Pad Prism software (version 5.0, San Diego, California USA). To identify significant effects between the treatments and control with or without stress conditions and endophyte, we used Duncan’s multiple range tests (DMRT) on Statistic Analysis System (SAS 9.1, USA).

## Results

### Co-synergism of endophyte and SA in plant biomass recovery under stress

**Figure 1:**
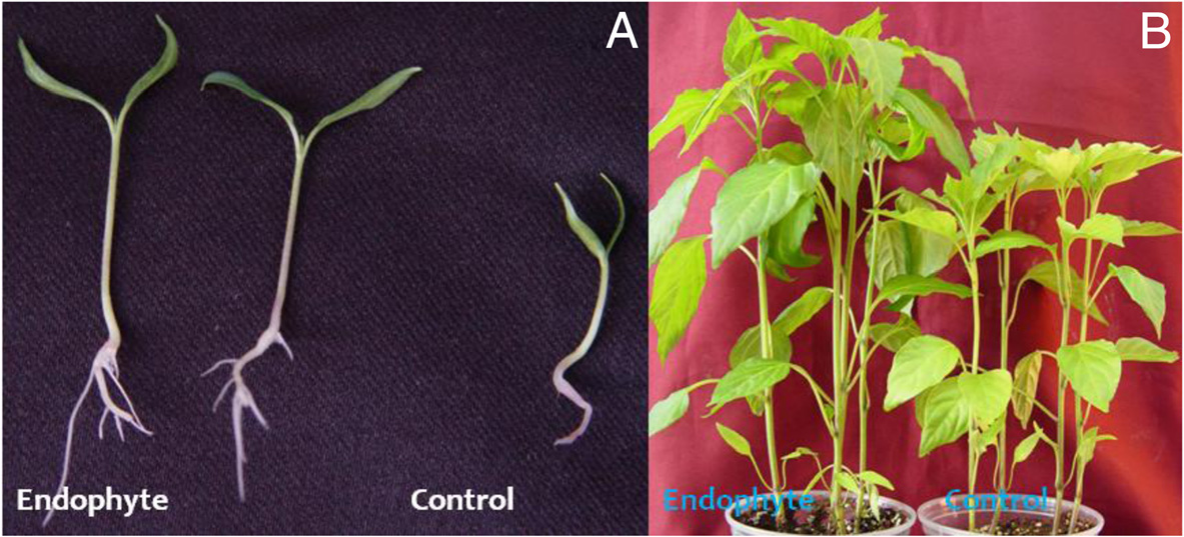
**Endophyte *P. resedanum *inoculated pepper (*Capsicum annuum *L.) plants growth after one week (A) and three weeks before stress (B).** Representative photo of pepper seedlings (18 per treatment) inoculated with or without endophytic fungi.

**Figure 2:**
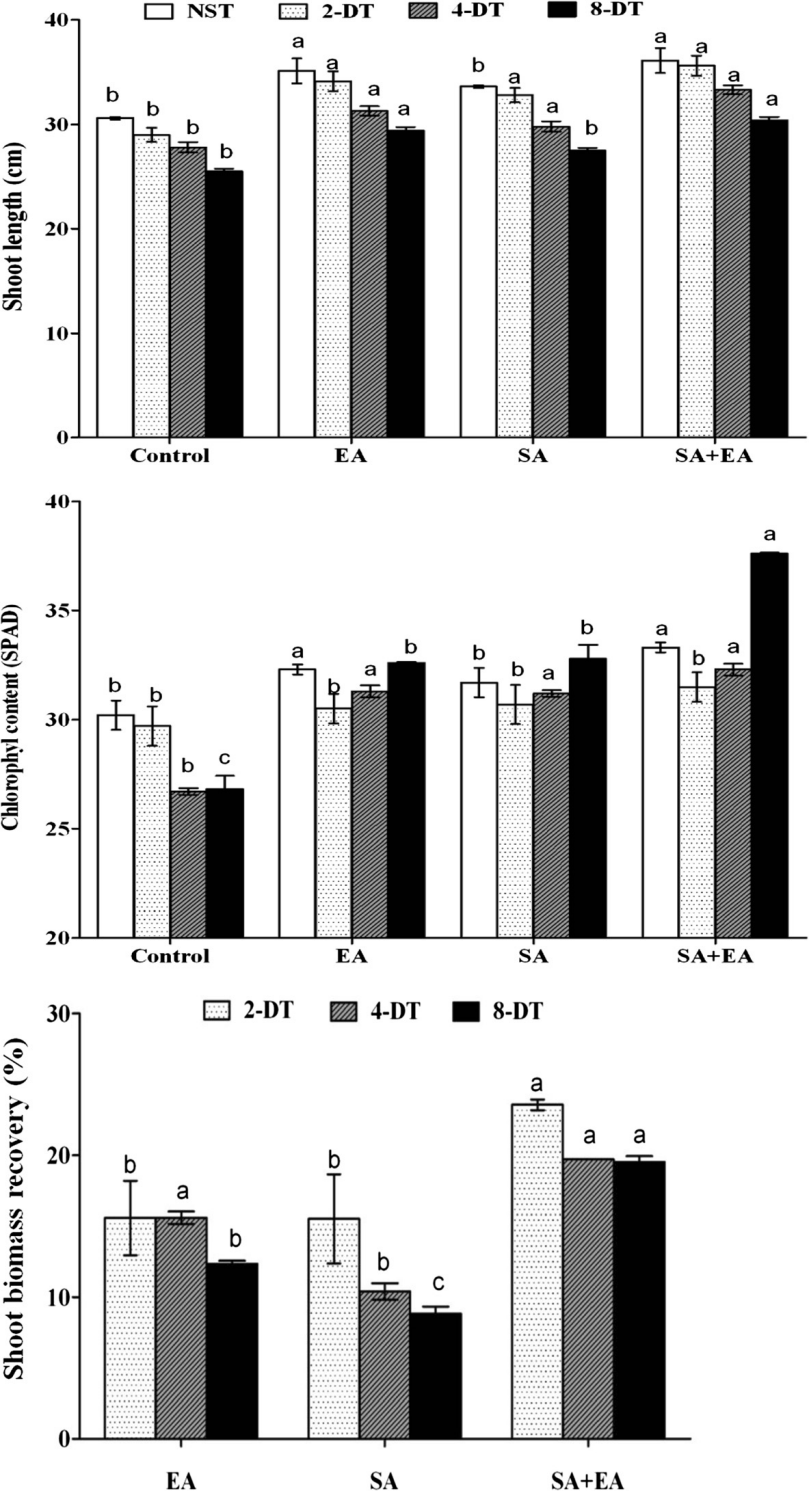
**Effects of endophyte *P. resedanum *association on the shoot length, chlorophyll content and shoot biomass recovery in various treatments after exposure to osmotic stress.** The control plants were treated with distilled water. EA plants were infected with *P. resedanum;* SA plants treated with 10^-6^ M SA, while SA+EA plants had endophytic-fungal association and treated with SA. 2-DT, 4-DT and 8-DT represent the osmotic stress induced by PEG for 2, 4 and 8 days respectively; NST (not stressed treatment). The different letter (s) in each treatment showed significant difference (*P*<0.05) as evaluated by DMRT.

The germinated pepper seeds were grown together with fungal endophyte *P. resedanum* (culture filtrate and mycelial propagules). After one week of endophyte association, the growth promoting effects were visible as compared to non-inoculated control plants. The endophyte-infected plants had higher growth rate and plant length than the control plants (Figure 1A). Similarly, when pepper plants were exposed to short-term drought stress for two, four and eight days, the shoot length was significantly reduced in non-infected control plants as compared to endophyte-elicited plants (Figure 1B and 2). With the increasing duration of drought stress, the plant height and metabolism reduced however, this was more pronounced in control than endophyte-inoculated plants. A similar growth dynamics was also shown by the exogenous application of SA with or without exposure to drought stress conditions (Figure 2). The endophyte-infected plants when applied with SA (with or without stress) resulted in significantly higher shoot length as compared to sole SA and control. Contrarily, the shoot lengths of plants inoculated with endophyte and treated with SA (SA+EA) and endophyte-associated (EA) plants were not significantly different from each other. It was observed that the non-inoculated plants without SA had significantly lower shoot lengths than the other three treatments i.e. EA, SA and SA+EA. Overall, the effect on shoot growth in EA and SA plants were not significantly different from each other. However, combination of SA+EA treatment exhibited significantly higher plant length as compared to other treatments.

The chlorophyll contents was higher in endophyte-infected plants than in non-infected plants. The drought-stress treated plants had significantly lower level of chlorophyll in non-inoculated plants whilst this was significantly higher in SA, EA and SA+EA plants during stress and normal growth conditions (NST). In control plants, the chlorphyl content was decreasing with the gradual exposure to drought (Figure 2). Conversely, in EA, SA and SA+EA plants, this trend was increasing with or without drought stress. It was significantly higher in SA+EA plants exposed to maximum duration of water deficient conditions. Beside this, we also observed that the photosynthesis rate was significantly higher in EA, SA and SA+EA plants.

**Figure 3:**
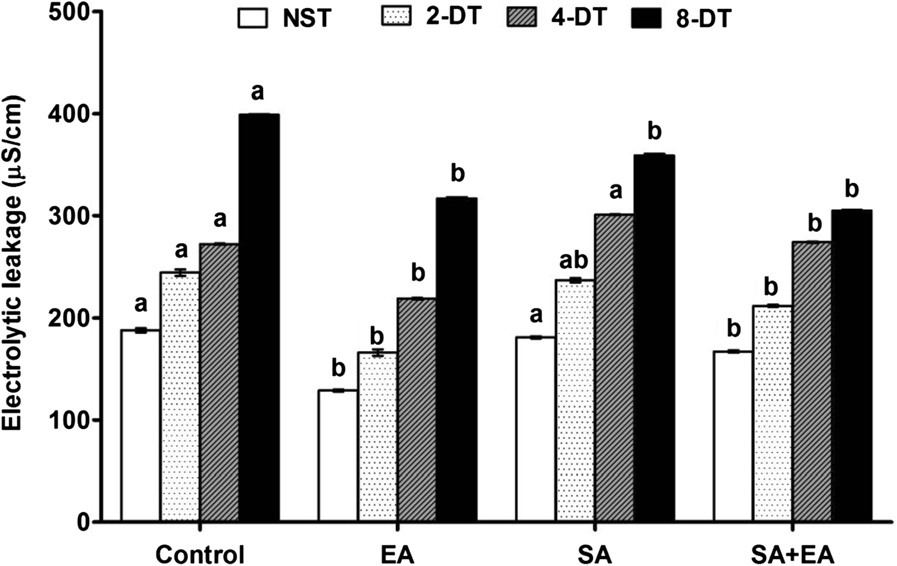
**Effect of endophyte symbiosis on the electrolytic release during stress.** EA = infected with *P. resedanum;* SA = treated with SA; SA+EA = endophytic-fungal associated plants treated with SA. NST (not stressed treatment), 2-DT, 4-DT and 8-DT represent drought stress period of 2, 4 and 8 days respectively.

The shoot length was 17.4, 13.3 and 23.3% higher in EA, SA and SA+EA treatments as compared to control after two days of stress. Similarly, after 4 and 8 days of stress, the shoot length increased 15.2, 10.8, 19.7% and 12.2, 9.1, 19.2% in EA, SA and SA+EA treatments respectively as compared to control (Figure 3). The biomass gains were prominent in the EA and SA+EA. During drought stress, the biomass loss was more prominent in control plants while our results did not shown significant difference between SA and EA plants (Figure 2).

Similarly, the plant biomass improvement during EA and SA+EA was also varified by the reduced electrolytic leakage (EL) in plants under stress. The results showed that EL was significantly higher in the non-inoculated control plants treated with 2, 4 and 8 days of drought. It was highly significant (*P*<0.001) in control after 8 days of stress (Figure 3). In comparison to sole SA-treated plants, the EL was lower than EA and SA+EA plants (Figure 3). The results suggest that the increased electrolytes influx represent higher tissue damages inside plants while this has been counteracted by the presence of endophyte with or without stress conditions.

**Figure 4:**
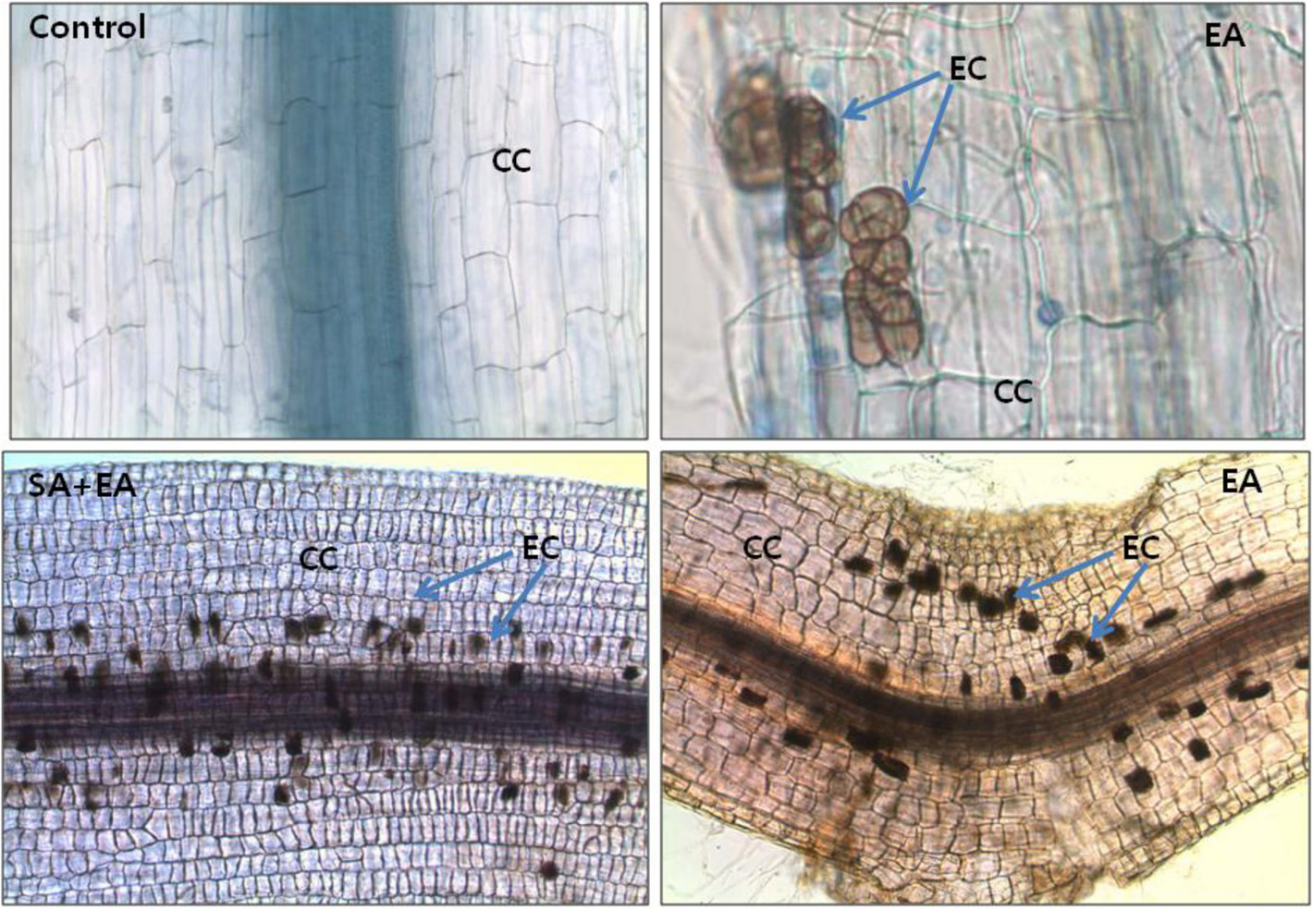
**Light micrographs of endophyte *P. resedanum – *associated with host plant’s root.** (Control) shows the light microscopic image of endophyte-free control plants (two weeks old). Bar = 200 μm. (EA) pepper root infected with *P. resedanum* after one week of inoculation. Microsclerotiums in the form of yeast-like cells were extensively colonized in the middle and inner cortex of the root. Bar = 40 μm; Bar = 100 μm. (SA+EA) presence of brownish yeast-like cells pericycle regions of the roots. Bar = 100 μm. The root samples were stain with tryptophan blue (0.8%). In the micrographs, CC = cortex cells; EC = endophyte cell.

The microscopic images showed the active association and habitation of *P. resedanum* inside the pepper plant’s root. The non-infected control plant’s roots were without any fungal association (Figure 4). The epidermal and cortex cellular region had no fungal infection. Contrarily, the microsclerotium of endophyte was seen in the inner cortex regions of the EA plant roots under normal growth conditions after one week of inoculation. However, endophyte colonization increased inside root with the passage of time and stress period. In SA+EA plants after 8 days of droughts stress, the rate of colonization was higher than the EA plants, suggesting that SA can also play an essential role in symbiotic microbial association (Figure 4).

### Antioxidant’s modulation during stress with *P. resedanum* and SA

**Figure 5:**
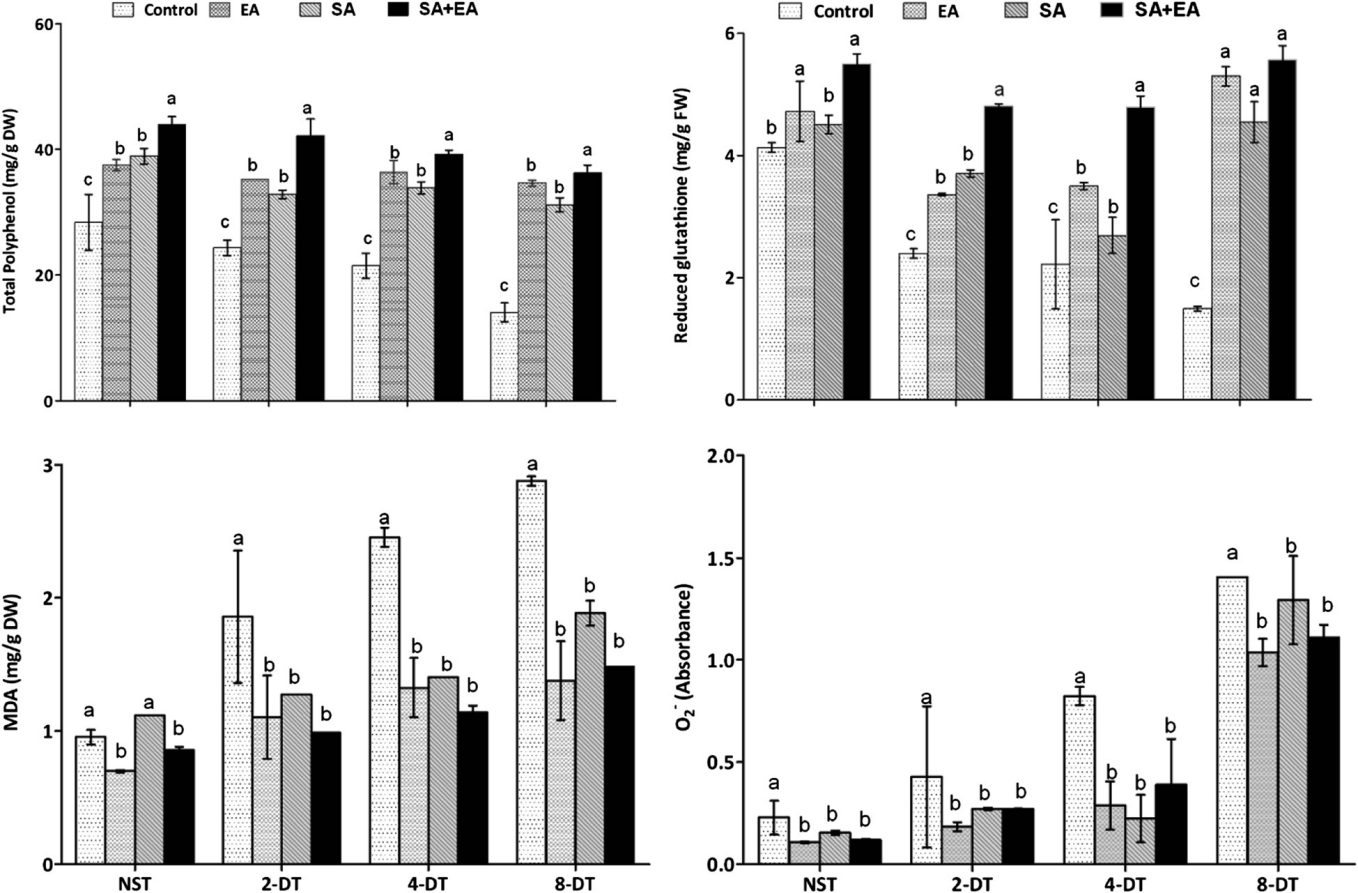
**Influence of drought stress on the antioxidants activities of the pepper plants inoculated with or without endophyte.** MDA refers to extent of lipid peroxidation; O_2_^-^ refers to superoxide anion. EA = infected with *P. resedanum;* SA = treated with SA; SA+EA = endophytic fungal associated plants treated with SA. NST, 2-DT, 4-DT and 8-DT represent non-stressed, 2, 4 and 8 days drought stressed plants respectively. The different letter(s) in each stress period showed significant difference (*P*<0.05) as evaluated by DMRT.

The results of antioxidant activities reveal stress modulation in pepper plants in the presence of endophyte as well as SA+endophyte under drought stress. The oxidative stress was promulgated by the imbalance in cellular water potential in control. In non-inoculated control, the total polyphenols were significantly lower than that of EA, SA and SA+EA treated plants. Though, the EA and SA plants had almost similar level of total polyphenol however in SA+EA plants, it was significantly higher. With immediate advent of stress conditions for two days, the total polyphenol level dropped down in non-inoculated plants as compared to other treatments like SA, EA and SA+EA treated plants. After 2 days of stress, endophyte-infested and SA treated plants have significantly higher total polyphenol levels as compared to sole EA and SA treated plants (Figure 5). Similarly, the increased osmotic stress in pepper further deteriorated the total polyphenol levels in control plants under 4 and 8 days of drought stress as compared to EA, SA and SA+EA plants. During high osmotic stress, the endophyte-associated plants maintained the total polyphenol level. We observed no significant different between EA, SA and SA+EA treated plants after exposure to 8 days of stress period.

Reduced glutathione (GSH) contents were significantly lower in control plants as compared to EA and SA+EA. The highest level of GSH formation was observed in SA+EA plants than other treatments. Upon osmotic stress, the GSH level reduced sharply in control plants as compared to other treatments (Figure 5). At 4^th^ and 8^th^ day of stress, the control and SA treated plant’s GSH level was lower than that of the EA and SA+EA plants. On 8^th^ day of stress, EA, SA and SA+EA plants were not significantly different in GSH level as compared to control plants. Thus, endophyte-association seems to have counteracted the stress in the presence of SA application.

The extent of lipid peroxidation (MDA content) was significantly regulated during the presence of endophytic-fungal association and SA application. The EA and SA+EA plant had lower level of MDA formation as compared control plants. SA and control plants, on the other hand, had no significant difference in MDA production under normal growth conditions. During osmotic stress, the MDA level in control plants increased abruptly from 2 to 8 days stress period. Conversely, this trend was significantly lower in SA, EA and SA+EA plants. Though, the MDA levels were high in SA treatments at the 8^th^ day of stress but this was significantly lower than that of control plants (Figure 5). Results suggest that the endophyte presence has counteracted drought stress by minimizing lipid peroxidation.

Supper oxide anions (O_2_-) were not significantly different between EA and SA plants. O_2_^-^ levels were higher in control plants under normal conditions. After the exposure to stress conditions (2 and 4 days), the O_2_^-^ formation was significantly high in control plants as compared to EA, SA and EA+SA plants. After 8^th^ day of stress, the O_2_^-^ levels were high in control and SA as compared to EA and SA+EA plants (Figure 5).

### Enzymatic regulation by endophyte and SA under stress

**Figure 6:**
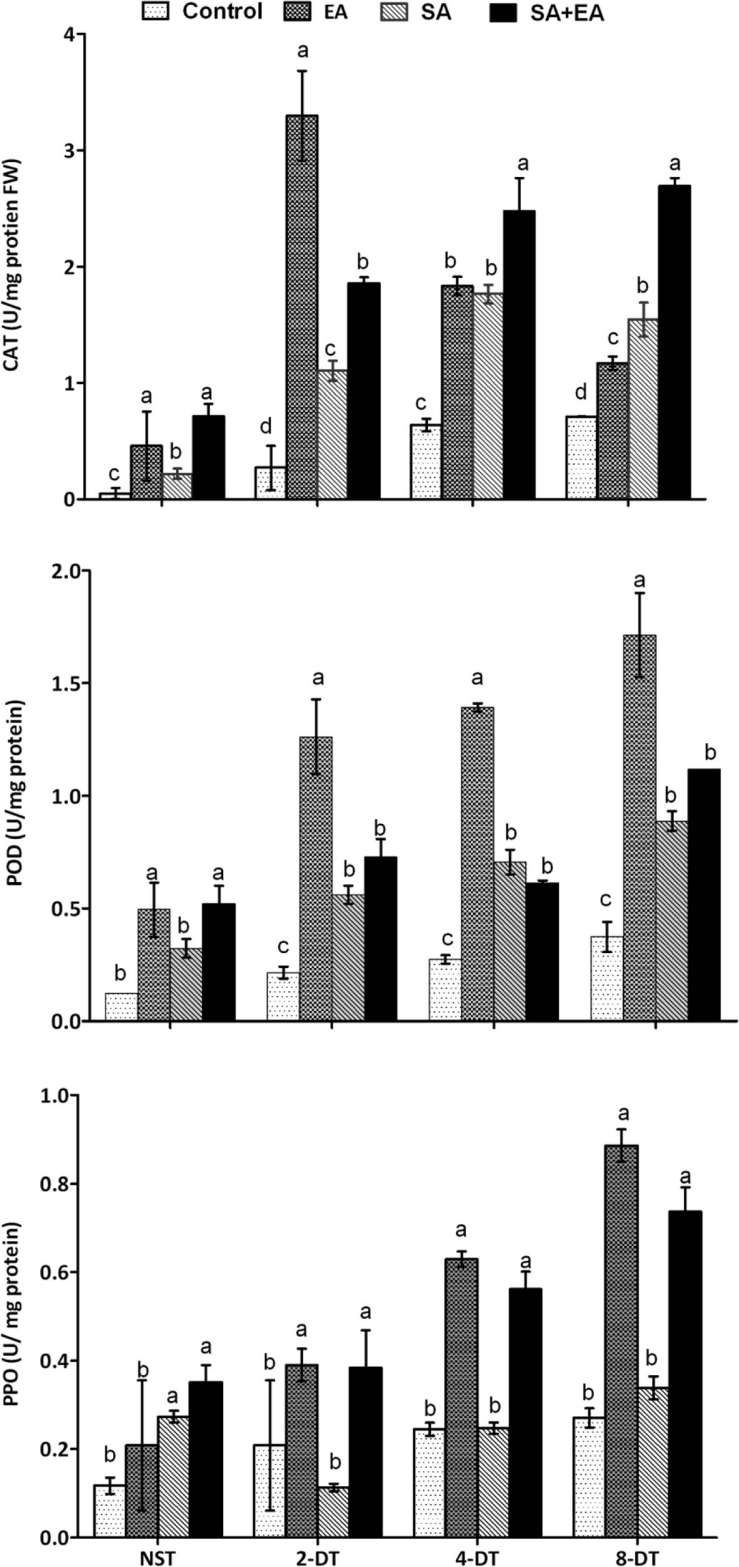
**Enzymatic activities of endophyte, SA and endophyte with SA treated plants during drought stress.** CAT = catalase; POD = peroxidase; PPO = polyphenol peroxidase. EA = infested with *P. resedanum;* SA = treated with SA; SA+EA = endophytic fungal associated plants treated with SA. NST, 2-DT, 4-DT and 8-DT represent non-stressed, 2, 4 and 8 days drought stressed plants respectively. The different letter(s) in each stress period showed significant difference (*P*<0.05) as evaluated by DMRT.

Enzymatic activities were significantly regulated during EA, SA and SA+EA. In catalase activity (CAT), it was significantly higher in EA and SA+EA plants while it was not significantly different between SA and control. In exposure to 2 days stress, the catalase activity was significantly activated in endophytic-associated plants as compared to control plants, SA and SA+EA plants. This activation trend was followed by SA+EA and SA plants respectively (Figure 6). In 4 and 8 days of stress, the catalase activity gradually reduced in EA plants whilst in SA and SA+EA it increased sharply. The catalase activity was significantly higher in SA+EA plants as compared to other treatments and control plants under maximum period of stress.

Peroxidase (POD) activities were significantly reduced in control plants with or without stress conditions as compared to other SA, EA and SA+EA plants. Under normal growth conditions, POD activity was significantly higher in EA and SA+EA plants as compared to SA plants (Figure 6). Upon exposure to osmotic stress for 2, 4 and 8 days, the POD activity was significantly higher in EA plants than SA and SA+EA plants and control plants. However, SA+EA plants had higher POD activity as compared to SA and control plants.

Polyphenol oxidase (PPO) activities were significantly reduced in control pepper plants. PPO activities increased in a dose dependent manner in EA plants with or without stress conditions. It was significantly higher in EA plants after 8 days stress (Figure 6). In SA treatments, PPO response with or without stress conditions was irregular. Although, PPO activity was comparatively lesser in SA+EA plants, it followed the same trend as we observed in EA plants.

### *P. resedanum* association and SA-dependent responses under abiotic stress

**Figure 7:**
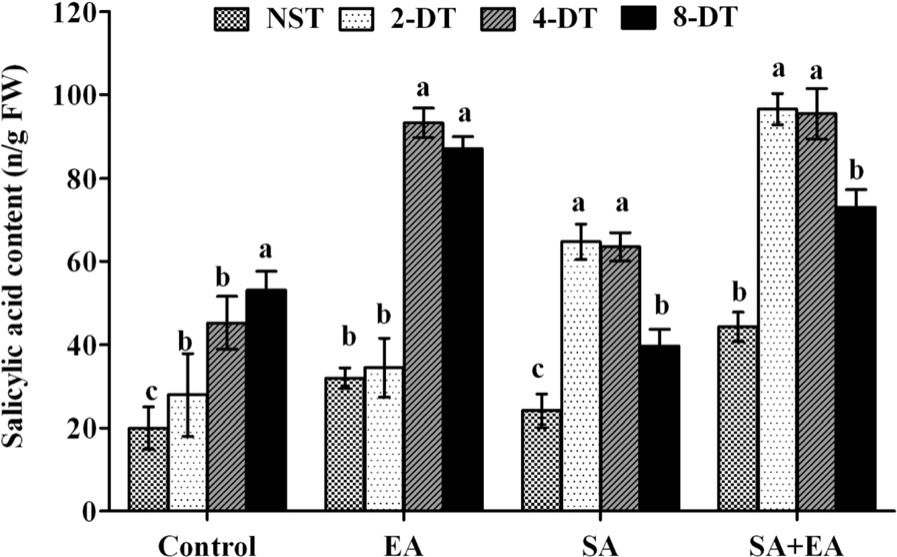
**Endogenous salicylic acid (SA) synthesis of pepper plants inoculated with or without *P. resedanum *under osmotic stress and normal growth conditions.** EA = infected with *P. resedanum;* SA = treated with SA; SA+EA = endophytic fungal associated plants treated with SA. NST, 2-DT, 4-DT and 8-DT represent non-stressed, 2, 4 and 8 days drought stressed plants respectively. The different letter (s) in each stress period showed significant difference (*P*<0.05) as evaluated by DMRT.

We also assessed the effect of endophytic elicitation with or without the treatment of SA on endogenous SA level. The results showed that SA was significantly low in non-stressed control. However, the stress periods has increased the endogenous SA levels (Figure 7). Similarly, in endophyte-associated plants, the endogenous SA was significantly higher than control under normal growth conditions. While after 2 days stress, its level in-significantly increased. The 4 and 8 days stress significantly increased SA contents in EA plants. This level was significantly higher than that of control and SA treated plants. In sole SA treatments, the plant synthesized low level of SA without any stress. However, upon 2 and 4 days stress, the SA level increased significantly while after 8 days, it decreased. In case of SA+EA plants, the endogenous SA followed the same trend as we noticed in sole SA treatments, however, the quantity of SA synthesized was significantly higher during similar conditions (Figure 7). The overall SA biosynthesis pathway activation in sole SA was lower than EA and SA+EA plants. The EA and SA+EA plants have significantly activated endogenous SA biosynthesis with or without stress conditions.

## Discussion

### Endophyte-association helps in biomass recovery

The results of the present study support and give additional information on the mechanism of endophyte’s ameliorative potential during abiotic stress to crop plant. The results revealed that endophyte-association rescued growth of pepper plants during stress by increasing shoot length. Plant-fungus relationship has been proclaimed a pivotal source for plant growth and development [30,31]. Endophytic fungi have been regarded as plant protectant and growth regulator during normal and extreme environmental conditions [15-20,31-33]. Various novel endophytic fungal species like *Piriformospora indica*, *Neotyphodium* sp., *Curvularia protuberate,* and *Colletotrichum* sp*.* etc [19,20,31,32,34] have been known to improve plant growth during abiotic stress conditions. *Penicillium* species have been known as a vital source for bioactive secondary metabolites [35]. Some strains of this genus also produce plant growth regulators like gibberellins, auxin, etc [17,19,33,36].

Gibberellins producing fungal genes cluster have been recently identified for *Phaeosphaeria* sp. L487 [37], *Gibberella fujikuroi*, *Sphaceloma manihoticola*[38] etc. Previous studies have shown that *Penicillium citrinum*[39], *P. paxilli*[40], *P. funiculosum*[17] produces gibberellins. It suggests the existence of GAs gene cluster in *Penicillium* spp.*;* hence, needs further genomic analyses at transcriptomics levels. In endophyte-host symbioses, consequences and interaction of secondary metabolites may be a contribution of the fungal endophyte to its host-plant to establish a mutualistic relationship [32,41]. Though, this process is very slow and the quantities of metabolites are very minute depending upon host and endophyte, but one way or the other, this barter trade always supports the host to counteract stress periods.

The phytohormones synthesis potential gives additional benefits to the host plants in mitigating the adverse affects of extreme environmental conditions salinity, drought and temperature stress as shown by Redman et al. [16], Khan et al. [17] and Hamilton and Bauerle [31]. Plants treated with the culture filtrate and propagules of endophytes are often healthier than endophyte-free ones [19,32]. Indeed, the endophyte-associations have enhanced biomass of barley [16], tomato [15], soybean [17] and rice [16] plants under various abiotic stress conditions like salinity, drought and high temperature. Pepper plants are adversely affected by abiotic stresses which retard their yield. It was observed that *P. resedanum* -associated plants had higher shoot length, chlorophyll content, and photosynthesis rate and low electrolytic leakages as compared to non-inoculated control. The non-inoculated plants, on the other hand, deprived of such association results in retarded growth and metabolism whilst they loss high plant biomass. This is also in conformity with the findings of Hamilton et al. [18] and Hamilton and Bauerle [31].

### ROS generation and oxidative stress modulation

It was found that the activities of antioxidants and related enzymes were significantly higher in endophyte-associated plants under osmotic imbalance induced ROS generation. With or without osmotic stress, endophyte elicitation has significantly regulated the antioxidant activities as compared to control and sole SA treated plants. It was shown that the responses of ROS generation and antioxidant signaling were similar to the effects caused by pathogenic and mutualistic microorganisms [42]. As both are different forms of consortiums however, higher antioxidant generation can improve plant defenses against disease and abiotic stress conditions. This was further elucidated by White and Torres [42] and Hamilton et al. [18]. Stress oriented ROS generations are minimized by the antioxidant and related enzymes production insides host-cells. ROS accumulation, on the other hand, has deleterious impacts on the functional membrane and macro-organelles. ROS are removed from the cell directly (catalase and peroxidase) or indirectly (redox molecules like glutathione).

The present findings showed higher levels of glutathione and total polyphenol and lower levels of lipid peroxidation and superoxide anion formation in the pepper plants associated with *P. resedanum*. The effects were more significant in SA+EA treated plants. It indicated that membrane injury was lower in endophyte-associated plants (EA and SA+EA) as the plants had lesser electrolytic leakage and lipid peroxidation (MDA content). Since membrane bounded lipid hydroperoxides are difficult to measure due to their instability, therefore we measured the degree of lipid peroxidation to quantify secondary breakdown products like MDA. Higher ROS, on the other hand, autocatalyze peroxidation of lipid membrane and affect membrane semi-permeability under high drought stress. Activation of antioxidant scavengers can enhance membrane stability against ROS attack while MDA content can be used to assess the stress injury of plants [43].

In stress related antioxidant enzymes, higher catalase (CAT), peroxidase (POD), and polyphenol oxidase (PPO) activities were observed in endophyte-infected plants as compared to non-infected control and sole SA-treated plants. CAT, POD and PPO have also been known to articulate the ROS induced oxidative burst. Increased catalase activity is associated with increased root length and enhanced seedling growth as shown by Harman [40]. Similarly, peroxidase and is polyphenol oxidase protects cells against the destructive influence of H_2_O_2_ by catalyzing its decomposition through the oxidation of phenolic osmolytes [44]. Previously, researchers have identified the crop growth regulation under stress conditions through activation of CAT, POD and PPO [20,31,45]. Similarly, the importance of endophyte colonization in terms of antioxidant activity and ROS production has been shown significance and often positive for the host-plant fitness [46], however this could be further verified by further experiments in case of *P. resedanum*.

### Co-synergism of SA with endophyte under osmotic stress

The SA application to the pepper plants had a growth promoting effect as compared to control plants. The SA also helped the plants to counteract the negative effects of osmotic stress. The effect of SA and EA on pepper shoot growth, chlorophyll contents was almost similar as compared to SA+EA treatments but this effect was significantly higher than control plants. Exogenous SA is known for its role in abiotic stress mitigation. In recent past, SA application has evidenced improved plant growth against abiotic stress [47-49]. Previous studies have shown that SA application to maize plant helped in alleviating the negative effects on the plants under drought stress [49]. Similarly, in case of tomato and bean plants, SA application has increased the plant biomass and decreased the drought induced oxidative stress by increasing the enzymatic activities [50]. Agarwal et al. [45] and Horvath et al. [9] also observed that SA application can improve plant biomass and enhance the antioxidant response against osmotic stress. The same is shown in our findings when we applied SA to pepper plants as compared to control plants.

During endophytic-fungal association, it was observed that the SA application to EA plants significantly increased the growth and metabolism as compared to sole SA and control plants. Furthermore, the biomass loss was much pronounced in non-inoculated and sole SA plants as compared to EA and SA+EA plants. Previously, it was shown that exogenous SA to roots of fungal-inoculated rice does not inhibit the root colonization of fungi [12]. Ludwig-Müller et al. [13] also reported that exogenous SA did not effected the root colonization by growth promoting fungi. However, our data shows the increased endophytic-colonization in SA treated host plants. This was also conformity to the results of Liu et al. [19], who indicated that exogenous SA application to fungal (*Glomus mosseae) inoculated Avena nuda* plants has increased the abiotic stress tolerance and had beneficial impacts on fungal colonization. The SA application to endophyte-inoculated plants not only increased endophytes abundance but also increased the host plant biomass, antioxidants and endogenous SA contents. It was shown that endogenous SA increased in endophyte-inoculated plants treated with SA as compared to sole SA and control plants under osmotic stress conditions.

Increased endogenous SA and antioxidant activities play an important role in abiotic and biotic defense signaling [47,48]. Under abiotic stress, high endogenous SA may mitigate the negative effects of ROS accumulation. Such functions can counteract the adverse effects of stress under mutualistic relationship as SA initiates induced systemic resistance [51]. Enhanced SA levels are especially important to reduce the susceptibility of plants to biotic and abiotic stresses [51]. We assume that the ISR stimulated through endophyte association activated the SA responses during osmotic stress. Mutualistic relationship initiates ISR and improves plant performance against biotic and abiotic stresses [43]. However, this concept is still overlooked in endophyte-induced ISR. Although *Penicillium* spp. have been known as potential inducers of ISR in various plants [11], our scientific understanding of the molecular mechanisms by which *Penicillium* sp. influence the outcome of plant abiotic stress tolerance is still marginal.

## Conclusion

Fungal endophyte, *P. resedanum* not only improves plant growth but also extend greater benefits to the host-plants to mitigate the negative effects of gradual osmotic stress. Exogenous SA application to pepper plant improved the stress tolerance of the plants while in combination with endophyte-inoculation it further regulated the stress impacts. The oxidative stress and cellular injury were reduced in the presence of endophyte and SA application. A significantly higher endogenous SA accumulation during endophytic fungal interaction and stress could be attributed to extend the tolerance against stress.

## Authors’ contributions

ALK planned and undertaken the research project. ALK performed the experiments, analyzed the data and drafted the manuscript. MH, MW and IJL had undertaken the plant hormonal work. AA and AA helped in revision of the MS and statistical analysis. All Authors contributed in writing the manuscript and approved its final content.

## Supplementary Material

Additional file 1 Table S1 HPLC conditions used for salicylic acid analysis. Click here for file
