# Supplementary material for: Co-synergism of endophyte Penicillium resedanum LK6 with salicylic acid helped Capsicum annuum in biomass recovery and osmotic stress mitigation
Source: BMC Microbiol. 2013 Mar 1;13:51. doi: 10.1186/1471-2180-13-51 (PMC3599947; doi:10.1186/1471-2180-13-51)
Supplement: Additional file 1 Table S1 — HPLC conditions used for salicylic acid analysis. [file 1471-2180-13-51-S1.doc]

**Supplementary Table 1.** HPLC conditions used for salicylic acid analysis.

| **Gradient** | **5min** | **2.5min** | **4.5min** | **5min** | **3min** |
| --- | --- | --- | --- | --- | --- |
| Solvent A | A : 30% | A : 40% | A : 60% | A : 30% | A : 30% |
| Solvent B | B : 70% | B : 60% | B : 40% | B : 70% | B : 70% |

Solvent A = 100% MeOH; Solvent B = 100% water in 0.5% acetic acid
